# Improvement of deep learning-based dose conversion accuracy to a Monte Carlo algorithm in proton beam therapy for head and neck cancers

**DOI:** 10.1093/jrr/rraf019

**Published:** 2025-04-23

**Authors:** Ryohei Kato, Noriyuki Kadoya, Takahiro Kato, Ryota Tozuka, Shuta Ogawa, Masao Murakami, Keiichi Jingu

**Affiliations:** Department of Radiation Physics and Technology, Southern Tohoku Proton Therapy Center, 7-172 Yatsuyamada, Koriyama, Fukushima, 963-8052, Japan; Department of Radiation Oncology, Tohoku University Graduate School of Medicine, 1-1 Seiryou-machi, Aoba-ku, Sendai, Miyagi, 980-8574, Japan; Department of Radiation Oncology, Tohoku University Graduate School of Medicine, 1-1 Seiryou-machi, Aoba-ku, Sendai, Miyagi, 980-8574, Japan; Department of Radiological Sciences, School of Health Sciences, Fukushima Medical University, 10-6 Sakaemachi, Fukushima, Fukushima, 960-8516, Japan; Department of Radiation Oncology, Tohoku University Graduate School of Medicine, 1-1 Seiryou-machi, Aoba-ku, Sendai, Miyagi, 980-8574, Japan; Department of Radiology, University of Yamanashi, 1110 Shimokato, Chuo-city, Yamanashi, 409-3898, Japan; Department of Radiation Physics and Technology, Southern Tohoku Proton Therapy Center, 7-172 Yatsuyamada, Koriyama, Fukushima, 963-8052, Japan; Department of Radiation Oncology, Tohoku University Graduate School of Medicine, 1-1 Seiryou-machi, Aoba-ku, Sendai, Miyagi, 980-8574, Japan; Department of Radiation Oncology, Southern Tohoku Proton Therapy Center, 7-172 Yatsuyamada, Koriyama, Fukushima, 963-8052, Japan; Department of Radiation Oncology, Tohoku University Graduate School of Medicine, 1-1 Seiryou-machi, Aoba-ku, Sendai, Miyagi, 980-8574, Japan

**Keywords:** proton therapy, deep learning, Monte Carlo, pencil beam algorithm, head and neck

## Abstract

This study is aimed to clarify the effectiveness of the image-rotation technique and zooming augmentation to improve the accuracy of the deep learning (DL)-based dose conversion from pencil beam (PB) to Monte Carlo (MC) in proton beam therapy (PBT). We adapted 85 patients with head and neck cancers. The patient dataset was randomly divided into 101 plans (334 beams) for training/validation and 11 plans (34 beams) for testing. Further, we trained a DL model that inputs a computed tomography (CT) image and the PB dose in a single-proton field and outputs the MC dose, applying the image-rotation technique and zooming augmentation. We evaluated the DL-based dose conversion accuracy in a single-proton field. The average γ-passing rates (a criterion of 3%/3 mm) were 80.6 ± 6.6% for the PB dose, 87.6 ± 6.0% for the baseline model, 92.1 ± 4.7% for the image-rotation model, and 93.0 ± 5.2% for the data-augmentation model, respectively. Moreover, the average range differences for R_90_ were − 1.5 ± 3.6% in the PB dose, 0.2 ± 2.3% in the baseline model, −0.5 ± 1.2% in the image-rotation model, and − 0.5 ± 1.1% in the data-augmentation model, respectively. The doses as well as ranges were improved by the image-rotation technique and zooming augmentation. The image-rotation technique and zooming augmentation greatly improved the DL-based dose conversion accuracy from the PB to the MC. These techniques can be powerful tools for improving the DL-based dose calculation accuracy in PBT.

## INTRODUCTION

In proton beam therapy (PBT), the dose-calculation accuracy in the human body is key to exploiting physical beam features, such as the Bragg peak. Presently, the analytical pencil beam (PB) algorithm, which can perform high-speed calculations, is employed for dose calculation in some PBT facilities, especially in those using a passive scattering PBT system. The PB algorithm computes the dose distribution in the patient for each narrow pencil beam using the measured depth dose and lateral scattering parameters [1]. As this algorithm can perform high-speed dose calculations, it is often employed for clinical dose calculations as well as optimization and robust evaluations in pencil beam scanning (PBS) [2]. However, the PB algorithm achieves decreased calculation accuracy in heterogeneous geometries, such as the lung or head and neck [3–6]. This decreased calculation accuracy prevents accurate range and Bragg peak estimations in a heterogeneous patient geometry, and this may affect the quality of PBT. Schuemann *et al.* revealed that the PB algorithm overestimates the dose by up to 5% in heterogeneous regions using a passive scattering proton beam [3]. Additionally, Yapes *et al.* reported that the PB algorithm overestimates the dose by >10% compared with the Monte Carlo (MC) algorithm in intensity-modulated proton therapy [5], and these dosimetric uncertainties may affect treatment outcomes [7]. Therefore, highly accurate dose-calculation algorithms, such as the MC algorithm, are required in heterogeneous regions, such as the lung or head and neck.

However, MC-based dose calculation is time-consuming, presenting a major clinical concern. To accelerate the MC calculation, methods based on graphic processing units (GPUs) [8, 9] as well as methods that handle only limited physical interactions [5, 10, 11] have been proposed. Several commercial treatment planning systems (TPS) have integrated the simplified MC calculation and are already achieving practical calculation times [12, 13]. However, the PB algorithm is still widely adopted as most users cannot implement the MC algorithm [2]. Although it is ideal for all facilities to quickly introduce the latest TPS, this may be impossible owing to economic factors. However, improving the dose calculation quality is crucial, even in such facilities. Moreover, passive scattering PBT systems are often not compatible with commercial MC. Therefore, high-quality dose calculation algorithms, such as MC algorithm, are desired regardless of the clinical environment, especially in passive scattering PBT systems.

In recent years, new approaches based on deep learning (DL) have been reported to accelerate MC calculation [14–20], exhibiting a potential to resolve the above issues. Nomura *et al.* and Wang *et al.* predicted proton three-dimensional (3D) dose distribution from patient geometries, contours, and beam spot information using DL [14, 15]. Javaid *et al.* employed DL to denoise the MC dose distribution with a small number of histories [16]. Moreover, Wu *et al.* calculated a dose distribution equivalent to an MC dose within a few seconds by converting the PB dose into the MC dose [17]. As any proton TPS can execute the PB algorithm, the DL-based dose conversion algorithm may be implemented at any proton treatment facility.

The performance of the DL model can be improved by introducing additional inputs as well as changing the loss function [15], boosting the neural network architecture [21, 22], and augmenting the training data, such as image rotation [19]. Some studies have attempted to train beams from the same direction to improve DL performance in PBT [17, 18]. Proton beams enter the patient from various directions and form dose distributions. Rotating the dose distribution with simple image processing creates a dose distribution virtually irradiated at the same angle [17]. We call this image processing image-rotation technique. Here, we hypothesized that the DL-based dose conversion accuracy from the PB dose to the MC dose could be further improved by exploring this image-rotation technique. This image-rotation technique may benefit the conversion scheme from a PB to an MC dose-calculation algorithm, as PBT is irradiated with fixed field ports regardless of the irradiation techniques, such as passive or scanning. However, the effectiveness of the image-rotation technique in ensuring DL accuracy in PBT has not been clarified. Therefore, we aimed to verify the impact of image rotation on the DL-based conversion scheme from the PB dose to the MC dose in passive scattering PBT for head and neck cancers. Although the need for the MC dose calculation for the lung region has been highlighted [7], MC dose calculations for the head and neck regions are also required. The head and neck regions are highly heterogeneous due to the air cavity and bone structure, and the organs at risk are often close to the target. Moreover, high doses are frequently prescribed for head and neck cancer therapy. Therefore, we believe that dose calculation errors can impose clinically significant risks. Furthermore, we hypothesized that data augmentation by zooming rotated images could increase the robustness of the DL model and improve the conversion accuracy from the PB dose to the MC dose in passive scattering PBT for head and neck cancers. Notably, no study has quantitatively evaluated the extent to which these approaches improve DL performance in PBT. In this study, we investigated the extent to which the model performance could be improved by data augmentation and evaluated their usefulness.

## MATERIALS AND METHODS

### Patient characteristics

We selected 85 patients with head and neck cancers who underwent PBT at our facility in 2022. This study was approved by the institutional review board of our facility. The number of beams in the dataset was 368, and the average number of beams per plan was 4. Their tumor locations included the oral cavity (34.8%), lymph node metastasis (23.2%), nasal cavity (22.3%), oropharynx (7.1%), hypopharynx (4.5%), parotid gland (4.5%), nasopharynx (1.8%), and larynx (1.8%). The average volume of clinical target volume (CTV) for all patients was 159.5 ± 210.4 cm^3^. In this study, the proton treatment plans were calculated by the PB algorithm on the proton TPS Xio-M (Hitachi, Kashiwa Japan). The dose distributions were calculated as a 2 × 2 × 2 mm grid. These dose distributions were exported for a single beam and used as input to the DL model.

### MC dose calculation

The MC calculation was based on Particle Therapy Simulation Framework (PTSIM) [23]. PTSIM is a Geant4-based radiotherapy-specific application. First, we modeled the nozzle structure, such as the wobbler electromagnet, scatterer, ridge filter, degrader, multileaf collimator, and range compensator, in the passive scattering proton therapy machine, Melthea (Hitachi, Kashiwa, Japan), at our facility to perform the MC dose calculations of the patient geometry using PTSIM. For head and neck cancers, we frequently employ the initial proton energy of 150 and 210 MeV. Thereafter, the energy, source position, and angular distribution were adjusted based on the measured mono-peak depth dose and spot size for the 150 and 210 MeV beams. As verifications, the depth doses and lateral profiles in water were compared for spread-out Bragg peak (SOBP) beams of 30 to 120 mm in 10-mm increments between the experimental and MC calculations using both 150 and 210 MeV beams. The SOBP widths and ranges between the experimental and MC calculations were within 2.5 and 0.7 mm, respectively, and the full width at half maximum and penumbra were within 1 mm. Additionally, we confirmed that the PB and MC dose calculations for several SOBP depth doses were within 2% and 3% in the SOBP and plateau regions, respectively.

To calculate the dose distribution in a heterogenous patient geometry using the MC algorithm, the material component and mass density must be estimated from a computed tomography (CT) image. We determined nine materials based on the CT values and mass densities using the conversion curve from the CT values to the stopping-power ratios registered in Xio-M [24]. Thus, our MC system calculated dose-to-medium for the same voxels as the PB algorithm in a heterogeneous patient geometry [25]. Moreover, we determined the beamline components, range compensator, multileaf collimator shape, and proton beam parameters based on the DICOM RT Plan exported from Xio-M. MC calculation was implemented on an Intel Xeon 5220R CPU with a 24-core processor system and processor base frequency of 2.2 GHz. For the simulation of a single field, we set proton histories from 2.5 × 10^8^ to 1.0 × 10^9^ according to the beam configuration. Furthermore, we allotted 6–24 CPU cores for the MC calculation of a single field depending on the total number of beams. Therefore, the calculation time was ~24 h per patient.

### DL architecture

In this study, we constructed a hierarchically dense (HD) U-net to convert the PB dose to the MC dose for each proton field. The HD U-net is a convolutional neural network developed by Nguyen *et al.* [26], it combines U-net [27] and DenseNet [28]. This architecture consists mainly three components: dense convolution, dense down sampling, and up-sampling. Each dense convolution part contained two dense convolution layers with a rectified linear unit (ReLU). Dense down sampling operation is performed by a stride convolution with ReLU and max pooling, and then these are concatenated. Finally, up-sampling part is done by up-sampling, dense convolution, and ReLU, followed by connecting the feature on the other side.

Xing *et al.* demonstrated the effectiveness of using a CT image as well as a dose distribution in the accurate dose conversion scheme for photon dose-calculation algorithms [29]. Therefore, we also added a CT image as an input for the conversion from the PB dose to the MC dose. This additional input allows the model to account for the heterogeneity that causes difference between the PB and MC dose. In this study, we verified the effectiveness of the image-rotation technique and data augmentation by image zooming in this DL-based conversion scheme.

### Data preprocessing

The patient dataset was randomly divided into 101 plans (334 beams) for training/validation and 11 plans (34 beams) for testing. All CT images and corresponding PB dose distributions for a single-field were interpolated and cropped to a size of 96 × 160 × 160, with a 2 mm voxel size at the isocenter as the origin. The CT values were rescaled between 0 and 1, and the PB and MC dose distributions were normalized by the maximum value of the PB dose for training the DL model.

**Figure 1:**
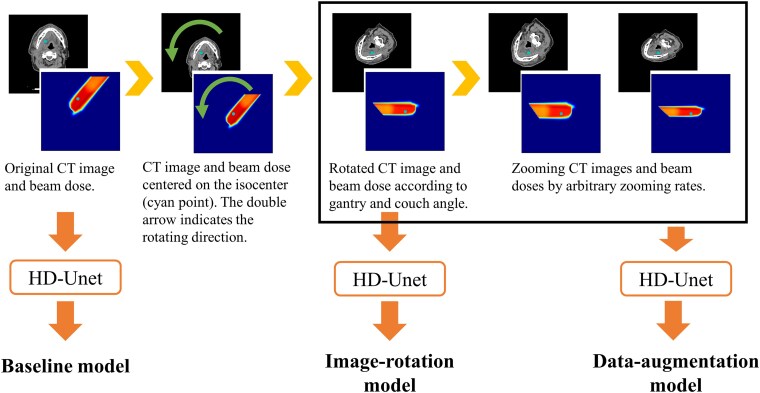
Workflow of the image-rotation and zooming-augmentation techniques. The point in the images indicates the isocenter. Also, the rotation arrow indicates the rotation direction according to the gantry angle.

Figure 1 shows an overview of the image rotation technique and zooming augmentation. In the image-rotation technique, the CT image and corresponding dose distribution were rotated around the isocenter as the beam was virtually irradiated gantry and couch angles of 270° and 0°, respectively. The default gantry and couch angle are arbitrary, and we selected 270° and 0°, respectively, for ease of programmatic processing. We often use non-coplanar beams for head and neck PBT to bring the treatment nozzle closer to the patient, and non-coplanar beams were 149 out of 368 beams in total. To increase the number of data and improve the robustness of the DL model, the rotated CT images and dose distributions for each beam were zoomed by 1.2 and 0.8 times in the training/validation datasets, respectively. Therefore, the number of training/validation datasets increased to 1002 cases. We tried the hyperparameter of the zooming rates between 0.7 and 1.3 where the dose distribution would not become unnatural, and we found that the zooming rates of 0.8 and 1.2 were suitable for our DL model. When converting the PB dose to the MC dose using the DL model on the test dataset, the output MC dose was rotated backward and interpolated based on the gantry and couch angles.

### Model training

Our DL model was implemented using Pytorch and trained on an NVIDIA A6000 GPU card with a memory capacity of 48 GB. The model was trained by the Adam optimizer, with an initial learning rate of 0.001, and if the validation loss did not improve for 10 epochs, it was decreased by 0.1. Furthermore, an early stopping technique with a patience level of 15 epochs was performed if the validation loss failed to improve. The DL model was trained for a maximum of 500 epochs, with a batch size of 4, using 3-fold cross-validation. We used a mean square error loss function to train the model.

### Evaluation of the accuracy of the DL model

We evaluated the performance of the DL model with the test dataset comprising 11 plans (34 beams). For the test data, the outputs from the three models that had been trained by 3-fold cross-validation were averaged for each single-field beam. To evaluate the effectiveness of the image-rotation technique and data augmentation by image zooming, we trained an HD U-net using the dataset without image rotation and data augmentation (baseline model). Furthermore, we constructed a comparison model using the dataset with the image-rotation technique only (image-rotation model). Afterward, we compared the performances of the model with image rotation and zooming augmentation (data-augmentation model) with other models.

The performances of the DL models were evaluated using the dose distribution for each single-field beam. Employing the dose for a single-field beam, we exactly evaluated the dose distribution. Moreover, we evaluated the plan dose that accumulated all beam doses because the total dose distribution is key to clinical applications.

### Evaluation for a single proton beam field dose distribution

**Figure 2:**
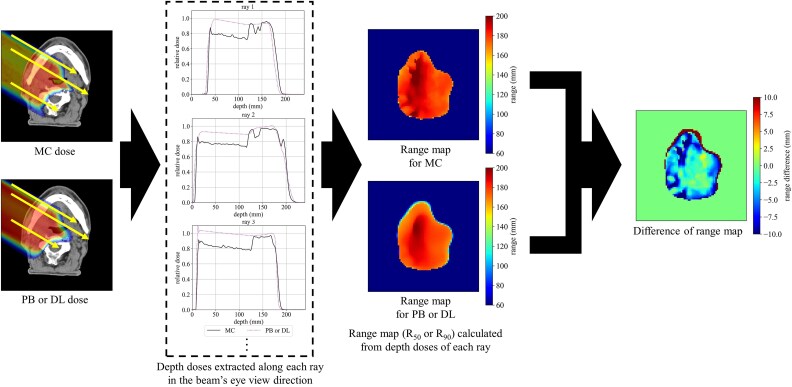
Overview of the range map calculated from the three-dimensional dose distribution.

To evaluate the agreement between the MC and DL doses in each DL model, we implemented a global 3D γ-analysis for each single-field beam with a criterion of 3%/3 mm and a threshold of 10% of the maximum MC dose in the body structure. We performed 3D γ-analysis using in-house Python program based on PyMedPhys library version 0.39.3. Furthermore, a range map was calculated from the dose distribution for each single-field beam, as range calculation is key to proton treatment planning. The range map extracted the range from the depth dose on the ray in the beam’s eye view direction [29]. Figure 2 shows the range extraction procedure. The range was defined as the distal fall-off position (R_90_), which is 90% of the prescribed dose for each beam, and R_50_. Although there are various definitions of range, we evaluated R_90_ and R_50_ as in the ref 29. From the two-dimensional range map of each ray, the average range difference (ARD) [29] in the MC and DL doses was calculated, as follows:


(1) \begin{equation*} ARD=\frac{1}{n}{\sum_{i=1}^n}\left({R}_{MC}(i)-R(i)\right)/{R}_{MC}(i) \end{equation*}

where n is the total number of pixels, ${R}_{MC}(i)$ is R_90_ or R_50_ of the i-th pixel of the range map in the MC dose, and $R(i)$ is R_90_ or R_50_ of the i-th pixel of the range map in the DL dose.

### Evaluation of the plan dose distributions

We also evaluated the performance of the DL model using the total dose distribution accumulated in all proton fields. First, the global γ-analysis with criteria 3%/3 mm, 2%/2 mm, and 1%/1 mm as well as a threshold of 10% was performed in the body contour of the test datasets. Second, the voxel-wise mean absolute error (MAE) in the body contour was evaluated for voxels ≥10% of the prescribed dose. MAE is calculated, as follows:


(2) \begin{equation*} MAE=\frac{1}{n}{\sum_{i=1}^n}\left|{D}_{MC}(i)-D(i)\right| \end{equation*}

Third, we calculated the dice similarity coefficient (DSC) in voxels ≥10% of the prescribed dose to evaluate the structural accuracy of the dose distribution from low to high dose regions. Finally, we evaluated doses receiving at least 98%, 95%, 50% and 2% of CTV (D_98%_, D_95%_, D_50%_, and D_2%_). The relative dose error (RDE) was calculated for each dose–volume metric, as follows:


(3) \begin{equation*} RDE=\frac{D-{D}_{MC}}{D_{MC}} \end{equation*}

## RESULTS

### Conversion time in deep learning model

The dose conversion from PB to MC doses was performed using NVIDIA A6000 GPU card with a memory capacity of 48 GB. The average conversion times for baseline model, image-rotation model, and data augmentation model were 0.24 ± 0.29, 0.23 ± 0.28, and 0.24 ± 0.30 sec, respectively.

### Evaluation of a single-field dose

**Figure 3:**
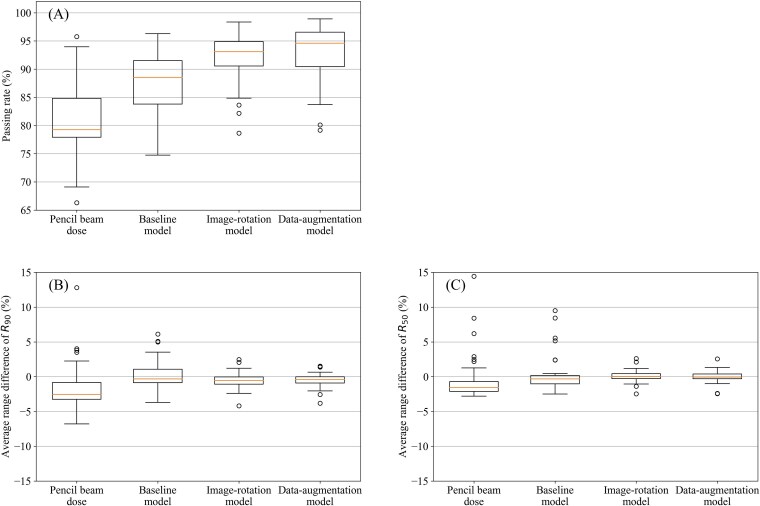
Boxplot of the evaluation in a single-field dose. (A) γ-passing rates with a criterion of 3%/3 mm, Mean absolute range difference for (B) R_90_ and (C) R_50_.

To evaluate the uncertainty of proton beams, the dose distribution for each single-field was compared by the global 3D γ-analysis with a criterion of 3%/3 mm. Figure 3A shows the γ-passing rate in the PB and DL doses. The average γ-passing rates for each single-field beam were 80.6 ± 6.6%, 87.6 ± 6.0%, 92.1 ± 4.7%, and 93.0 ± 5.2% for the PB dose, baseline model, image-rotation model, and data-augmentation model, respectively. Moreover, the γ-passing rate in the DL dose was evidently improved by the image-rotation technique, and further improved by the zooming augmentation.

Figure 3B shows the ARDs of R_90_. The mean ARDs of R_90_ were − 1.5 ± 3.6%, 0.2 ± 2.3%, −0.5 ± 1.2%, and − 0.5 ± 1.1% for the PB dose, baseline model, image-rotation model, and data-augmentation model, respectively. Furthermore, Fig. 3C shows the ARDs of R_50_. The mean ARDs of R_50_ were − 0.3 ± 3.6%, 0.4 ± 2.7%, 0.1 ± 0.9%, and 0.0 ± 0.9% for the PB dose, baseline model, image-rotation model, and data-augmentation model, respectively. Employing the image-rotation technique, the ARDs were greatly improved compared with those for the PB.

### Evaluation of the plan dose

**Figure 4:**
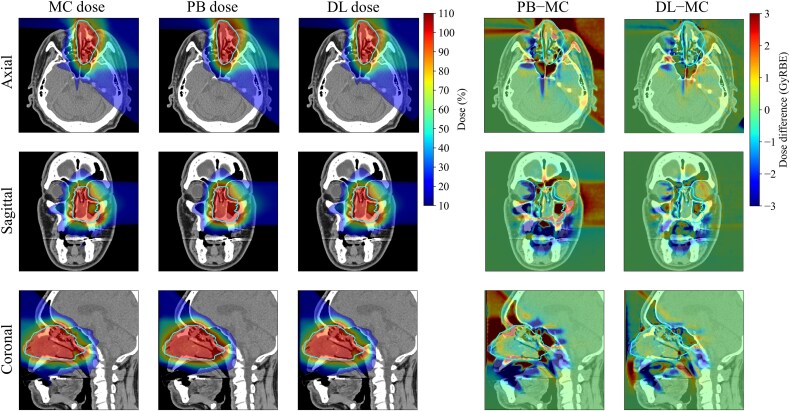
Example of the dose distributions for each calculation algorithm. The contour indicates the CTV. The DL dose shows the data-augmentation model.

**Figure 5:**
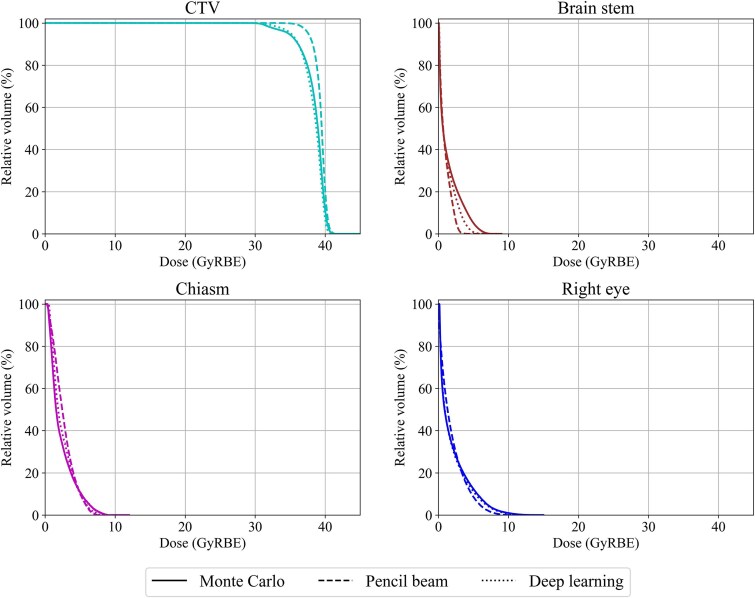
Dose–volume histogram of a patient in Fig. 4. The solid, dashed, and dotted lines represent the Monte Carlo algorithm, pencil beam algorithm, and our developed deep learning-based algorithms (the data-augmentation model), respectively.

We also evaluated the dose distribution for the clinical plan, which accumulated all proton beam fields. Figure 4 shows an example of the dose distribution calculated using each algorithm in the test dataset. This patient underwent a PBT boost after volumetric modulated arc therapy at our facility. The CTV in this case was in the nasal cavity, which is a heterogeneous region containing a mixture of air, bone, and soft tissues. Compared with the ground-truth MC dose, the PB dose exhibited large calculation errors on the distal end whereas the DL dose exhibited a good agreement with the MC dose. The DL dose was greatly improved compared with the PB dose, especially in the air cavity. The shape of the dose–volume histogram (DVH) for the CTV in the DL dose was more similar to that in the MC dose than that in the PB dose (Fig. 5). Moreover, the DVH shape for risk organs, such as the brain stem, in the DL was close to that in the MC dose.

Table 1 shows the average γ-passing rates for the plan dose in the testing dataset. The 3D γ-passing rates in the DL dose were greatly improved compared with those in the PB dose. Employing the image-rotation technique, a high passing rate (average 92.7 ± 2.7%) with a criterion of 2%/2 mm was achieved compared with that in the baseline model. Employing the zooming augmentation, the γ-passing rate was further improved by an average of 93.9 ± 3.3%.

**Table 1:** Comparison results between each dose distribution and the Monte Carlo dose distribution

	γ-passing rate			MAE (GyRBE)
	1%/1 mm	2%/2 mm	3%/3 mm	
Pencil beam dose	54.4 ± 8.2%	78.1 ± 7.2%	89.5 ± 4.8%	1.30 ± 0.39
Baseline model	64.7 ± 9.9%	86.6 ± 6.0%	94.8 ± 3.3%	0.93 ± 0.27
Image-rotation model	73.6 ± 6.4%	92.7 ± 2.7%	97.9 ± 1.2%	0.68 ± 0.18
Data-augmentation model	75.8 ± 6.6%	93.9 ± 3.3%	98.3 ± 1.4%	0.64 ± 0.19

The average MAEs in the body contour was 1.30 ± 0.39 GyRBE, 0.93 ± 0.27 GyRBE, 0.68 ± 0.18 GyRBE, and 0.64 ± 0.19 GyRBE for the PB dose, baseline model, image-rotation model, and data-augmentation model, respectively (Table 1). The DL dose combined with the image-rotation technique and the zooming augmentation greatly improved the absolute dose error compared with the PB dose.

To investigate the structural accuracy, the DSCs in the isodose regions between the MC dose and each dose distribution were calculated. Table 2 shows the average DSC in the PB or DL doses. In the DL doses, all average DSCs were greatly improved compared to the PB dose, and the average DSCs in the data-augmentation model were the highest. Moreover, the data-augmentation model achieved the average DSCs of 0.9 even in the low dose regions, exhibiting the best performance in DL-based dose conversion.

**Table 2:** Dice similarity coefficient of the isodose volumes above 10% of the prescription dose

	Pencil beam dose	Baseline model	Image-rotation model	Data-augmentation model
10%–30%	0.810 ± 0.067	0.842 ± 0.063	0.891 ± 0.035	0.902 ± 0.032
10%–50%	0.889 ± 0.037	0.912 ± 0.031	0.939 ± 0.017	0.945 ± 0.015
10%–70%	0.915 ± 0.022	0.934 ± 0.018	0.955 ± 0.009	0.959 ± 0.008
10%–90%	0.930 ± 0.015	0.945 ± 0.014	0.962 ± 0.008	0.965 ± 0.007

Table 3 shows the average RDEs for the CTV calculated using the PB or DL doses. The dose–volume metrics of D_98%_ and D_95%_ were improved by the image-rotation technique than the PB dose and were further improved by combining image-rotation technique with zooming augmentation. Almost no change in D_50%_ and D_2%_ in any DL model was comparable with those in the PB dose.

**Table 3:** Dose–volume metric errors for clinical target volume

	Relative dose error			
	D_98%_	D_95%_	D_50%_	D_2%_
Pencil beam dose	3.7 ± 5.9%	2.1 ± 3.8%	0.1 ± 0.9%	−0.3 ± 1.6%
Baseline model	2.2 ± 1.6%	1.7 ± 1.2%	1.3 ± 1.4%	0.3 ± 1.8%
Image-rotation model	0.5 ± 1.8%	0.2 ± 1.0%	−0.2 ± 0.9%	−1.1 ± 1.3%
Data-augmentation model	0.0 ± 1.6%	−0.3 ± 1.0%	−0.5 ± 1.1%	−1.2 ± 1.1%

## DISCUSSION

We attempted to further improve the conversion accuracy from the PB dose to the MC dose by DL for PBT using the image-rotation technique and data augmentation by image zooming. Our results confirmed that the γ-passing rates and range estimations in each proton beam field were improved by image rotation and that the performance was further improved by zooming augmentation. As shown in Fig. 4, the DL dose improved considerably compared with the PB dose, particularly in the air cavity. This indicates that the use of the DL-based dose conversion can correct for the dose differences due to material handling in the dose calculation algorithms. By learning the same angular dose distributions using the image rotation technique, the DL model can efficiently extract the difference between PB and MC doses. Further, data augmentation by image zooming improves the robustness of DL-based dose conversion accuracy. If there are a few training datasets, the number of datasets can be increased by varying the zooming rates. These are very useful techniques for DL-based dose conversion from PB to MC dose.

PBT exhibits physical advantages, such as a range, which is used to form a unique dose distribution. Therefore, the accuracy of range estimation is key to proton treatment planning. The accuracy of the PB algorithm is limited, particularly in heterogeneous regions [3–6]. Thus, an accurate dose-calculation algorithm, such as the MC algorithm, is required. DL is among the known methods for solving the calculation time issue in MC dose calculation [14–20]. Wu *et al.* demonstrated that the DL-based dose conversion time is approximately a few seconds [17]. The dose conversion time was ~0.2 sec for the three DL models evaluated in our study. Therefore, DL considerably reduces the time required to calculate the MC-equivalent dose. The extant studies mostly evaluated dose distributions that were predicted or calculated by DL using plan doses; they did not examine the range. In our study, we strictly evaluated the uncertainties of the dose and range in each proton beam field. The mean ARDs for R_90_ and R_50_ were improved in the DL dose compared to the PB dose. As the data-augmentation model did not further improve the range uncertainty, it is sufficient to estimate it with only the image-rotation technique. An evaluation of the range revealed that the range as well as dose distribution was improved in the conversion scheme from the PB dose to the MC dose using DL.

We considered the possible implementation of the PB dose calculation in any proton TPS, and adopted the same DL architecture as that of Wu *et al.* to convert the PB dose to the MC dose [17]. They trained the DL model on other treatment sites, such as the lung, liver, and prostate, as well as the head and neck, and achieved an average γ-passing rate of 92.8% (1%/1 mm) for the testing dataset of head and neck. Their γ-passing rates with 1%/1 mm were considerably different from our results. However, their head and neck cancer training data may be biased. We believe that the main reason for this difference is the diversity of the training data, as our DL model was trained using data on various types of head and neck cancers. Furthermore, the direct comparison of our results with those of previous studies was challenging because of various factors, such as the irradiation methods, reported dose settings (dose-to-water or dose-to-medium), adjustment methods of the MC platform, planning policies, and variation of training data. However, we aimed to verify the effectiveness of the image-rotation technique and zooming augmentation in the DL-based dose conversion schema. By evaluating the dose distribution and range of each proton beam field, we demonstrated the effectiveness of these techniques. Moreover, we comprehensively evaluated the dose distribution accumulated in all proton fields by 3D γ-analysis, MAE, isodose DSC, and RDE, and demonstrated the effectiveness of our methods.

The PB dose-calculation algorithm is still widely employed in some proton TPS, regardless of the irradiation methods, such as passive scattering or PBS [2]. Furthermore, some proton TPSs implement the MC dose-calculation in PBS systems, thereby achieving the practical calculation speed and accuracy [12, 13]. Although the transition from the passive scattering system to PBS is advancing worldwide, some facilities still provide passive scattering PBT. Notably, not all proton TPSs are also compatible with the MC dose-calculation. Therefore, some PBT facilities may delay the transition to the clinical application of the MC dose-calculation algorithm. Our facility is one of the facilities that do not have the MC dose-calculation algorithm installed in the TPS. Thus, we have constructed a secondary check system for TPS using PTSIM. However, it is challenging to use it daily in clinical treatment planning owing to the extremely long calculation time (approximately several hours). Further, updating the current TPS to the latest TPS with the MC dose-calculation algorithm installed may be challenging for economic reasons. To resolve these issues, we considered the conversion scheme from the PB dose to the MC dose using DL. Although commissioning is required to implement this DL-based conversion schema in clinical practice, any proton therapy facility can rapidly calculate dose distributions comparable to the MC algorithm using the DL-based approach. Therefore, the conversion scheme from the PB dose to the MC dose developed in this study is highly beneficial. Moreover, this approach may apply to dose calculations in passive scattering PBT and robust evaluation and optimization in PBS, as well as even adaptive PBT. We believe that the DL-based dose conversion schema is a novel approach to boost the proton treatment planning accuracy.

We acknowledged several limitations to our study. In the range evaluations, we observed no small difference even using the DL model. The reason for these errors may be the diversity of the training datasets. Further strengthening of the DL model will require more accurate proton range estimation. Moreover, we developed the DL model for a passive scattering PBT system. In recent years, PBS has become the mainstream technology for PBT, and the number of facilities using PBS is increasing globally. Therefore, our DL model may exhibit more application potential in PBS systems than in passive scattering systems. Wu *et al.* adapted a DL model developed with a passive scattering system to a PBS using transfer learning [17]. As we use the same DL architecture as them, we can immediately adapt our DL model to the PBS system by the same transfer learning. Moreover, we developed DL-based dose conversion model for head and neck only. PBT is performed other tumor sites, such as lung, liver, and prostate, as well as head and neck. Thus, we plan to develop the DL-based dose conversion model adopted for various tumor sites in the future.

In conclusion, we verified the effectiveness of the image-rotation technique and data augmentation by image zooming in improving the conversion accuracy from the PB dose to the MC dose using DL. The image-rotation technique decreased the uncertainties in the dose distribution and proton range for each proton beam field. Furthermore, zooming augmentation further improved the γ-passing rates. Therefore, preprocessing by combining image rotation and zooming augmentation can very effectively improve the dose conversion accuracy from the PB dose to the MC dose. The DL-based dose-calculation accuracy and prediction can be further improved by integrating our techniques into proton TPS and that dose conversion scheme can provide accurate clinical treatment plans for any PBT facility.

## PRESENTATION AT A CONFERENCE

Partial results of this study were presented in the 127^th^ Scientific Meeting of Japan Society of Medical Physics.

## References

[1] Hong L, Goitein M, Bucciolini M et al. A pencil beam algorithm for proton dose calculations. Phys Med Biol 1996;41:1305–30. 10.1088/0031-9155/41/8/005. 8858722

[2] Teoh S, Fiorini F, George B et al. Is an analytical dose engine sufficient for intensity modulated proton therapy in lung cancer? Br J Radiol 2020;93:20190583. 10.1259/bjr.20190583. 31696729 PMC7066954

[3] Schuemann J, Giantsoudi D, Grassberger C et al. Assessing the clinical impact of approximations in analytical dose calculations for proton therapy. Int J Radiat Oncol Biol Phys 2015;92:1157–64. 10.1016/j.ijrobp.2015.04.006. 26025779 PMC4509834

[4] Yamashita T, Akagi T, Aso T et al. Effect of inhomogeneity in a patient's body on the accuracy of the pencil beam algorithm in comparison to Monte Carlo. Phys Med Biol 2012;57:7673–88. 10.1088/0031-9155/57/22/7673. 23123683

[5] Yepes P, Adair A, Grosshans D et al. Comparison of Monte Carlo and analytical dose computations for intensity modulated proton therapy. Phys Med Biol 2018;63:045003. 10.1088/1361-6560/aaa845. 29339570 PMC5906701

[6] Paganetti H, Jiang H, Parodi K et al. Clinical implementation of full Monte Carlo dose calculation in proton beam therapy. Phys Med Biol 2008;53:4825–53. 10.1088/0031-9155/53/17/023. 18701772

[7] Taylor PA, Kry SF, Followill DS. Pencil beam algorithms are unsuitable for proton dose calculations in lung. Int J Radiat Oncol Biol Phys 2017;99:750–6. 10.1016/j.ijrobp.2017.06.003. 28843371 PMC5729062

[8] Giantsoudi D, Schuemann J, Jia X et al. Validation of a GPU-based Monte Carlo code (gPMC) for proton radiation therapy: clinical cases study. Phys Med Biol 2015;60:2257–69. 10.1088/0031-9155/60/6/2257. 25715661 PMC7788741

[9] Jia X, Schümann J, Paganetti H, Jiang SB. GPU-based fast Monte Carlo dose calculation for proton therapy. Phys Med Biol 2012;57:7783–97. 10.1088/0031-9155/57/23/7783. 23128424 PMC4474737

[10] Fippel M, Soukup M. A Monte Carlo dose calculation algorithm for proton therapy. Med Phys 2004;31:2263–73. 10.1118/1.1769631. 15377093

[11] Kohno R, Takada Y, Sakae T et al. Experimental evaluation of validity of simplified Monte Carlo method in proton dose calculations. Phys Med Biol 2003;48:1277–88. 10.1088/0031-9155/48/10/303. 12812446

[12] Lin L, Huang S, Kang M et al. A benchmarking method to evaluate the accuracy of a commercial proton Monte Carlo pencil beam scanning treatment planning system. J Appl Clin Med Phys 2017;18:44–9. 10.1002/acm2.12043. PMC5689961 28300385

[13] Liang X, Li Z, Zheng D et al. A comprehensive dosimetric study of Monte Carlo and pencil-beam algorithms on intensity-modulated proton therapy for breast cancer. J Appl Clin Med Phys 2019;20:128–36. 10.1002/acm2.12497. PMC6333133 30488548

[14] Nomura Y, Wang J, Shirato H et al. Fast spot-scanning proton dose calculation method with uncertainty quantification using a three-dimensional convolutional neural network. Phys Med Biol 2020;65:215007. 10.1088/1361-6560/aba164. 32604078

[15] Wang W, Chang Y, Liu Y et al. Feasibility study of fast intensity-modulated proton therapy dose prediction method using deep neural networks for prostate cancer. Med Phys 2022;49:5451–63. 10.1002/mp.15702. 35543109 PMC10358316

[16] Javaid U, Souris K, Huang S, Lee JA. Denoising proton therapy Monte Carlo dose distributions in multiple tumor sites: a comparative neural networks architecture study. Phys Med 2021;89:93–103. 10.1016/j.ejmp.2021.07.022. 34358755

[17] Wu C, Nguyen D, Xing Y et al. Improving proton dose calculation accuracy by using deep learning. Mach Learn Sci Technol 2021;2:015017. 10.1088/2632-2153/abb6d5. 35965743 PMC9374098

[18] Neishabouri A, Wahl N, Mairani A et al. Long short-term memory networks for proton dose calculation in highly heterogeneous tissues. Med Phys 2021;48:1893–908. 10.1002/mp.14658. 33332644

[19] Spezialetti M, Lapenna F, Caianiello P., et al. Using deep learning for fast dose refinement in proton therapy. 2021 IEEE International Conference on Systems, Man, and Cybernetics (SMC). Honolulu, HI: IEEE, 2021, 1783–1789. 10.1109/SMC52423.2021.9658879.

[20] Pastor-Serrano O, Perkó Z. Millisecond speed deep learning based proton dose calculation with Monte Carlo accuracy. Phys Med Biol 2022;67:105006–6. 10.1088/1361-6560/ac692e. 35447605

[21] Osman AFI, Tamam NM. Attention-aware 3D U-net convolutional neural network for knowledge-based planning 3D dose distribution prediction of head-and-neck cancer. J Appl Clin Med Phys 2022;23:e13630. 10.1002/acm2.13630. 35533234 PMC9278691

[22] Aso T, Kimura A, Kameoka S et al. GEANT4 based simulation framework for particle therapy system. IEEE Nuclear Science Symposium Conference Record 2007;4:2564–7. 10.1109/NSSMIC.2007.4436673.

[23] Chang W, Koba Y, Furuta T et al. Technical note: validation of a material assignment method for a retrospective study of carbon-ion radiotherapy using Monte Carlo simulation. J Radiat Res 2021;62:846–55. 10.1093/jrr/rrab028. 33998654 PMC8438268

[24] Paganetti H . Dose to water versus dose to medium in proton beam therapy. Phys Med Biol 2009;54:4399–421. 10.1088/0031-9155/54/14/004. 19550004

[25] Ronneberger O, Fischer P, Brox T. U-Net: Convolutional Networks for Biomedical Image Segmentation. Springer International Publishing, Springer, LNCS, 2015. 234–41. 10.1007/978-3-319-24574-4_28.

[26] Nguyen D, Jia X, Sher D et al. 3D radiotherapy dose prediction on head and neck cancer patients with a hierarchically densely connected U-net deep learning architecture. Phys Med Biol 2019;64:065020. 10.1088/1361-6560/ab039b. 30703760

[27] Huang G, Liu Z, Van Der Maaten L et al. Densely connected convolutional networks. Proceedings of the IEEE conference on computer vision and pattern recognition. Honolulu, HI: IEEE, 2017:2261–9.

[28] Xing Y, Zhang Y, Nguyen D et al. Boosting radiotherapy dose calculation accuracy with deep learning. J Appl Clin Med Phys. 2020;21:149–59. 10.1002/acm2.12937. 32559018 PMC7484829

[29] Schuemann J, Dowdell S, Grassberger C et al. Site-specific range uncertainties caused by dose calculation algorithms for proton therapy. Phys Med Biol 2014;59:4007–31. 10.1088/0031-9155/59/15/4007. 24990623 PMC4136435

